# Bi‐directional, long‐distance hormonal signalling between roots and shoots of soil water availability

**DOI:** 10.1111/ppl.13697

**Published:** 2022-05-20

**Authors:** Katharina Huntenburg, Jaime Puértolas, Carlos de Ollas, Ian C. Dodd

**Affiliations:** ^1^ Lancaster Environment Centre Lancaster University Lancaster UK; ^2^ NIAB Agronomy NIAB Cambridge UK; ^3^ Department of Botany and Plant Ecology and Physiology University of La Laguna San Cristóbal de La Laguna Spain; ^4^ Departamento de Ciencias Agrarias del Medio Natural Universitat Jaume I Castellon Spain

## Abstract

While the importance of plant water relations in determining crop response to soil water availability is difficult to over‐emphasise, under many circumstances, plants maintain their leaf water status as the soil dries yet shoot gas exchange and growth is restricted. Such observations lead to development of a paradigm that root‐to‐shoot signals regulate shoot physiology, and a conceptual framework to test the importance of different signals such as plant hormones in these physiological processes. Nevertheless, shoot‐to‐root (hormonal) signalling also plays an important role in regulating root growth and function and may dominate when larger quantities of a hormone are produced in the shoots than the roots. Here, we review the evidence for acropetal and basipetal transport of three different plant hormones (abscisic acid, jasmonates, strigolactones) that have antitranspirant effects, to indicate the origin and action of these signalling systems. The physiological importance of each transport pathway likely depends on the specific environmental conditions the plant is exposed to, specifically whether the roots or shoots are the first to lose turgor when exposed to drying soil or elevated atmospheric demand, respectively. All three hormones can interact to influence each other's synthesis, degradation and intracellular signalling to augment or attenuate their physiological impacts, highlighting the complexity of unravelling these signalling systems. Nevertheless, such complexity suggests crop improvement opportunities to select for allelic variation in the genes affecting hormonal regulation, and (in selected crops) to augment root–shoot communication by judicious selection of rootstock–scion combinations to ameliorate abiotic stresses.

## 1 INTRODUCTION

Once plants evolved to abandon the water environment and colonise the land surface, they faced the problem of capturing carbon dioxide from the air. Maintaining gas exchange required an ability to transport water from where it is available (soil) to the shoots, and to regulate transpirational losses to avoid desiccating in a dry atmosphere. Therefore, plants developed a complex network of signals acting on the stomata that regulate shoot gas exchange according to soil water availability and atmospheric evaporative demand.

Signals perceived by roots and transmitted to above‐ground organs (mainly leaves) have long been proposed, and thoroughly reviewed elsewhere (Li et al., 2021). Changes in soil water potential are transmitted hydraulically along the soil‐to‐leaf continuum, with decreased leaf water potential and turgor declining concomitantly with soil water availability, thereby limiting cell division and expansion and inducing stomatal closure (Hsiao, 1973). Loss of turgor pressure of stomatal guard cells reduces their volume and hence stomatal aperture. Long‐distance hydraulic signals of soil drying regulating shoot physiology was the dominant paradigm over many decades. However, different controlled environment experimental systems induced soil drying without altering leaf water potential, by implementing heterogeneous soil drying in split‐root systems, pneumatically pressurising the roots, or growing plants in large soil columns that allowed plant root systems to explore similar soil volumes as in the field while maintaining low evaporative demand around the shoots (reviewed in Dodd, 2005). Thus, the paradigm shifted from an exclusively hydraulic long‐distance signal in response to soil drying to the proposition that root‐sourced chemical signals transported in the xylem sap could also induce stomatal closure, with most attention focused on abscisic acid (ABA) in view of its potent antitranspirant action.

Nevertheless, ABA's role as a necessary long‐distance signal of soil drying has been questioned since wild‐type (WT) plants grafted on ABA‐deficient rootstocks show normal stomatal closure as the soil dries (Holbrook et al., 2002). Moreover, reciprocal grafting experiments with WT and ABA‐deficient mutants demonstrated that shoots regulate root ABA accumulation (McAdam, Brodribb, & Ross, 2016). These experiments provided an alternative paradigm, focusing attention on foliar ABA synthesis or redistribution causing stomatal closure and acting as a shoot‐to‐root ABA signal that causes root ABA accumulation (Castro et al., 2019). Nevertheless, although recent evidence does not support a main role of root‐sourced ABA in shoot responses to soil drying, the previously mentioned observations and new evidence from other hormone groups suggest that root‐sourced hormones can modulate the physiological responses mediated by leaf‐sourced ABA.

Considering the importance of ABA in regulating plant water relations, it should not be surprising that plants have redundant long‐distance signalling systems allowing acclimation responses to soil drying in distal organs, depending on whether the roots or shoots are the first to lose turgor in response to soil drying and/or high evaporative demand. We discuss the role of these signalling systems by reviewing the evidence for root ABA synthesis, xylem transport and its effects on shoot physiology, along with the effects of basipetal phloem transport of ABA. Furthermore, we consider other hormones (jasmonates and strigolactones [SL]) implicated in stomatal regulation to evaluate whether plants also adopt these hormones in root‐ and shoot‐sourced signalling of drying soil, and interactions between these hormonal signalling systems and ABA. While additional signalling systems such as mobile peptides and reactive oxygen species (ROS) also seem important in communicating soil water status, the reader is directed to other sources that cover non‐hormonal signals (Li et al., 2021).

## 2 ABSCISIC ACID

Long‐distance signalling conceptually involves three conditions. First, localised environmental changes (e.g. drought) must stimulate signal production in the emitting organ. Secondly, the signal should be transmitted from the emitting to the receiving organ and finally, the signal must trigger physiological processes in the receiving organ. Since roots are in contact with soil, usually they are the first to perceive soil drying and early long‐distance signalling (whether hydraulic or chemical) of drought must be elicited in this organ.

### 2.1 Root ABA synthesis in drying soil?

Blocking phloem transport by girdling (surgical removal of phloem tissue at the root–shoot junction) suppressed soil drying‐induced ABA accumulation in roots (Castro et al., 2019; Manzi et al., 2015), suggesting negligible root–autonomous ABA synthesis. However, earlier studies demonstrate substantial ABA accumulation in detached, air‐dried roots, indicating roots have the capacity to synthesise ABA (Borel et al., 2001; Simonneau et al., 1998). ABA is biosynthesised from C_40_ carotenoids in plastids and limited availability of these precursors can reduce ABA levels (Borel et al., 2001). Even though carotenoid synthesis is much greater in chloroplasts, water and osmotic stress in roots enhance the expression of enzymes involved in ABA and carotenoid biosynthesis in root plastids, which are enhanced by ABA itself in a positive feedback mechanism needed for stress‐induced ABA synthesis (Ruiz‐Sola et al., 2014). Thus, the root possesses mechanisms to synthesise not only ABA, but also its precursors. Nevertheless, since carotenoid synthesis, which is derived from the MEP pathway, ultimately requires availability of primary metabolites such as pyruvate or glyceraldehyde‐3‐phosphate as precursors, blockage of photosynthates transport from shoots impairs ABA synthesis. This explains observations that girdling limited root ABA accumulation (Castro et al., 2019), even though de‐topped roots severed from the shoot for a long period (3 weeks) still upregulated these carotenoid and ABA biosynthesis genes when transferred to dry soil (Manzi et al., 2016). Moreover, the hypothesis that root ABA synthesis does not contribute to root ABA accumulation is difficult to reconcile with differential ABA accumulation in dry and wet parts of the root‐zone under heterogeneous soil drying (Puértolas et al., 2015). Thus, while recent results seem to indicate that root‐autonomous ABA synthesis makes a negligible contribution to root ABA accumulation, there is ample evidence that soil drying can increase root ABA synthesis.

Nevertheless, the roots may generate other signals before ABA, such as sulphate ions (Malcheska et al., 2017) or small peptides (Takahashi et al., 2018), which upregulate NCED (9‐*cis* epoxy carotenoid dioxygenase—a key ABA biosynthesis enzyme) gene expression in the shoot. In considering whether ABA acts as a long‐distance signal of drying soil, a key question is whether the ABA synthesised de novo in the roots and transported to the shoot can affect shoot physiology by partially closing the stomata or inhibiting leaf growth.

### 2.2 Does root‐synthesised ABA affect shoot responses?

Root xylem sap ABA concentration is often better correlated with stomatal closure than bulk leaf ABA levels (Castro et al., 2019) and naturally occurring xylem sap ABA concentrations can induce stomatal closure in some species when supplied via the xylem to detached leaves (Rothwell et al., 2015). However, other results questioned whether drought‐induced xylem ABA concentrations are sufficient to induce partial stomatal closure or inhibit leaf growth (Munns & Cramer, 1996). Also, a split‐root experiment with reciprocal grafts between WT and ABA‐deficient mutants showed that the high root xylem ABA concentration produced by WT rootstocks could not revert the ‘wilty’ phenotype of ABA‐deficient scions, while WT scions closed their stomata even when grafted on ABA‐deficient rootstocks (Holbrook et al., 2002 Figure 1A,C,D). This evidence suggested the existence of other root‐to‐shoot signals that can modulate ABA action regulating stomatal closure in response to soil drying.

**FIGURE 1:**
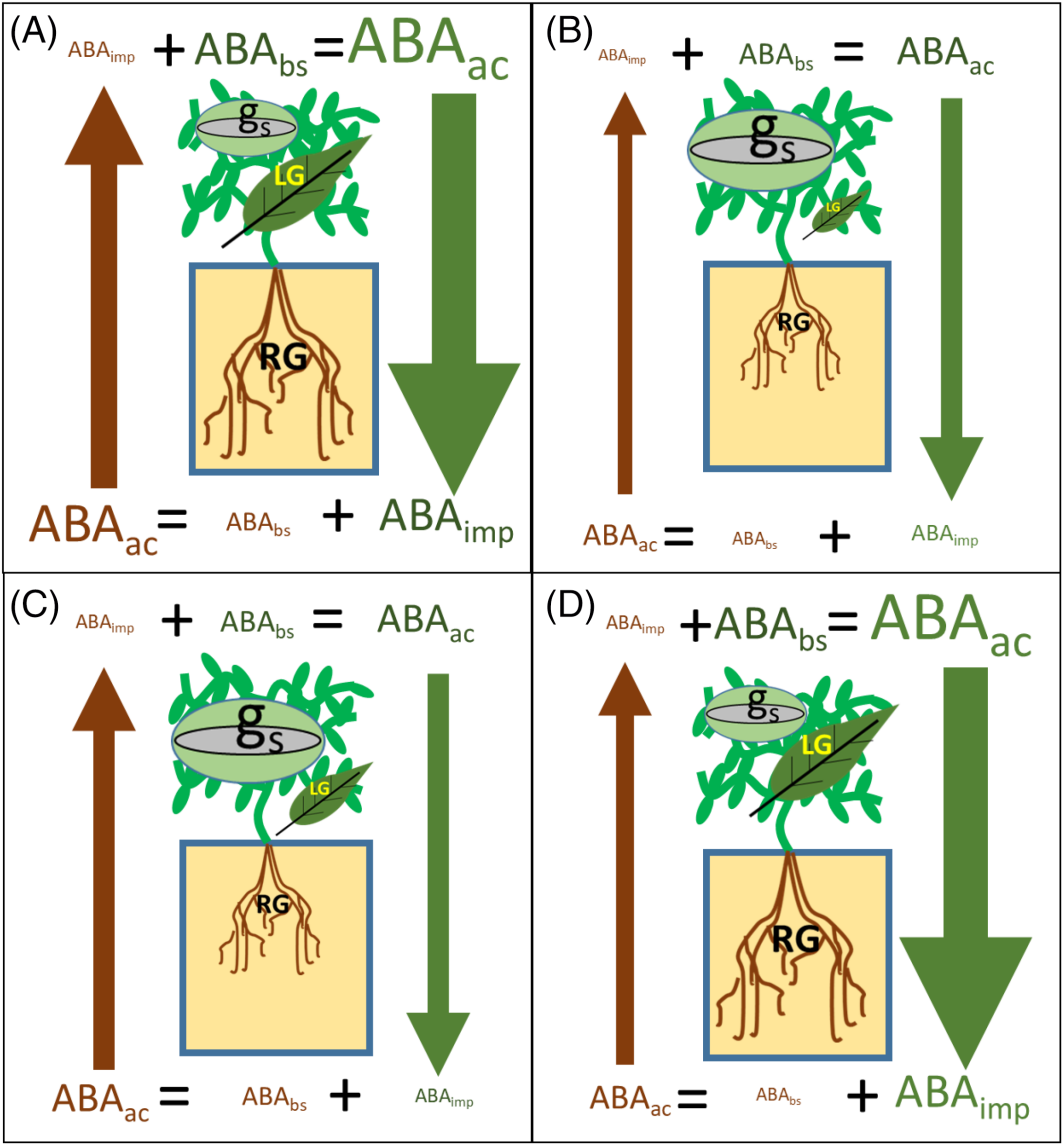
Schematic representation of the effects on stomatal conductance (*g*_s_), leaf growth (LG) and root growth (RG) of different combinations of ABA‐deficient (*ABA*) and wild‐type (WT) genotypes in reciprocal grafting experiments. Letter and symbol size represents relative magnitude of the variables (compared across different panels). ABA_imp_, abscisic acid imported to the organ (green = shoot, brown = root); ABA_bs_, abscisic acid biosynthesised in the organ; ABA_ac_, total ABA accumulated in the organ. Briefly, compared with WT/WT self‐grafts (A), ABA‐deficient self‐grafts ABA/ABA (B) have reduced ABA_bs_ in both organs (shoot and root) and hence ABA traffic between them, consequently reducing ABA_ac_, which results in less LG and RG and higher *g*_s_. Root ABA_bs_ in aba/WT (scion/rootstock) grafting (C) is similar to WT/WT but lower ABA_imp_ from shoots reduces root ABA_ac_ and RG to only slightly higher values than aba/aba plants (McAdam, Brodribb, & Ross, 2016). Increased ABA_imp_ from roots to shoots compared with aba/aba does not increase shoot ABA_ac_ so *g*_s_ phenotype is similar (Holbrook et al., 2002, but see Li et al., 2018 and references therein), while LG is partially restored as root‐to‐shoot ACC signalling is attenuated (Dodd et al., 2009). Finally, ABA_imp_ into roots in WT/aba plants (D) restores RG to similar levels as WT/WT (McAdam, Brodribb, & Ross, 2016), while reduced ABA_imp_ into shoots does not make any substantial impact on shoot phenotype compared with WT/WT (Holbrook et al., 2002).

Xylem sap alkalisation in response to soil drying was proposed as a long‐distance signal enhancing partitioning of ABA to the leaf apoplast in contact with the guard cells and away from the mesophyll (Wilkinson & Davies, 1997). The bundle sheath cell proton pump, AHA2 plays a key role in regulating xylem sap pH and leaf radial hydraulic conductance of *Arabidopsis* (Grunwald et al., 2021). However, xylem sap alkalisation is not a universal response to soil drying in all species. Other changes in xylem sap composition were also identified as early signals of soil drying that promoted stomatal closure (Malcheska et al., 2017; Sobeih et al., 2004; Visentin et al., 2016) suggesting that ABA seems not to act as the earliest or primary actor of root‐to‐shoot signalling of soil drying.

Nevertheless, the ability of a WT rootstock to phenotypically revert the ABA status and physiology of ABA‐deficient scions varied substantially between ostensibly similar studies (Li et al., 2018 and references therein). Whether xylem ABA transport increases leaf ABA concentration may depend on the rate of ABA metabolism in the shoot (Trejo et al., 1995). Other environmental stresses such as increased atmospheric evaporative demand, which elicits ABA synthesis in the leaf (McAdam, Sussmilch, & Brodribb, 2016), may also alter the relationship between root ABA export and foliar ABA accumulation. Plants grown in conditions that reduce foliar ABA synthesis capacity, like low vapour pressure deficit (VPD) levels (Giday et al., 2014) may be more reliant on root ABA synthesis to regulate stomatal responses to soil drying, as the relative importance of root‐sourced ABA on shoot ABA accumulation could be greater.

The role of root‐to‐shoot ABA signalling in controlling shoot growth is even less clear. Nevertheless, a WT rootstock substantially increased leaf area of an ABA deficient scion by decreasing its xylem sap ACC concentration and leaf foliar ethylene evolution (Dodd et al., 2009, Figure 1B,C). Similar grafting experiments under typical Mediterranean greenhouse conditions demonstrated that root‐supplied ACC was negatively correlated to tomato vegetative biomass while root‐supplied ABA was positively correlated to vegetative biomass (Cantero‐Navarro et al., 2016). Furthermore, ABA overexpressing rootstocks increased reproductive growth of wild‐type tomato scions grown at moderate salinity (3.5 ds m^−1^) via an interaction with gibberellin levels (Martínez‐Andújar et al., 2021). Thus, root‐supplied ABA may be more important in regulating shoot growth than leaf gas exchange, with these responses being modulated by other phytohormones.

### 2.3 Shoot‐to‐root ABA signalling

Although water and osmotic stresses promote root ABA synthesis, the shoots can produce much more ABA with some of it transported to roots via phloem. ABA, in interaction with other hormones such as auxins and ethylene, is essential in regulating root growth (Li et al., 2017). By decreasing root levels of the auxin 3‐indoleacetic acid (IAA), shoot‐sourced ABA may promote root growth (Figure 1C,D) and inhibit lateral root formation (McAdam, Brodribb, & Ross, 2016). Moreover, when Citrus plants were grown under low evaporative demand and the roots allowed to dry, root gene expression of carotenoid biosynthesis enzymes was responsive to leaf but not root rehydration (Manzi et al., 2017). In contrast, dehydrated detached roots of the same species expressed those carotenoid synthesis genes (Manzi et al., 2016), suggesting that other shoot‐sourced signals repress ABA synthesis in the roots when leaf water potential remains high. Shoot‐to‐root flows of ABA could not only regulate root growth and function but re‐distribute shoot‐sourced ABA. Girdling experiments indicate that when ABA cannot be transported to the roots, it accumulates in shoots inducing stomatal closure even in well‐watered plants (Castro et al., 2019).

Thus shoot‐to‐root ABA signalling is not only an important mechanism regulating root growth under water deficit, but also highlights the role of ABA in the complex bi‐directional communication between roots and shoots (Figure 1). Further research on this topic should disentangle the influence of different above‐ and below‐ground environmental cues on the relative importance of these communication mechanisms. In addition, the extensive research carried out in past decades on root‐to‐shoot ABA signalling in response to heterogeneous soil drying must be re‐examined by considering these bi‐directional signalling systems.

### 2.4 Jasmonates

Jasmonates are a family of molecules derived from fatty acid metabolism and structurally related to the hormone jasmonic acid (JA), including the JA biosynthetic precursor 12‐oxophytodioneic acid (OPDA) and JA derivatives like methyl jasmonate (MeJA) and (3R,7S)‐jasmonyl‐l‐isoleucine (JA‐Ile). Among other intermediates and precursors in JA biosynthesis with possible bioactive properties, JA‐Ile is considered the bioactive molecule as it has the strongest interaction with the JA receptor COI (Wasternack, 2014). Following lipoxygenase (LOX) activity on α‐linoleic acid to initiate jasmonates biosynthesis, OPDA is the last jasmonate synthesised in the plastid by allele oxidase synthase (AOS) and allele oxide cyclase (AOC) and then transported to the peroxisome where 12‐oxo‐phytodienic acid reductase3 (OPR3) generates an intermediate that is transformed into JA after three cycles of non‐enzymatic β oxidation. The jasmonate resistant 1 enzyme (JAR1) catalyses the last step to synthesise JA‐Ile.

### 2.5 Drying soil and biosynthesis of jasmonates

Jasmonates are directly or indirectly involved in multiple biotic and abiotic stresses and are considered a “master switch” in many plant stress responses in shifting from growth to defence and activating the synthesis of multiple secondary metabolites. Similar to ABA, JA concentration is higher in the shoots than roots (Castro‐Valdecantos et al., 2021), since most of their biosynthetic route occurs in chloroplasts. JA was proposed to operate as a paracrine signal capable of being transported from cell to cell in tomato leaves (Farmer & Ryan, 1992). The phloem mobile JA precursor OPDA can also promote stomatal closure in *Arabidopsis*, with increased OPDA content related to decreased stomatal aperture and improved drought resistance (Savchenko et al., 2014). Thus, JA and OPDA can act as drought stress signals in the shoot.

Osmotic stress (10% and 20% PEG‐6000) rapidly (within 3 h) upregulated root expression of the cotton JA biosynthesis genes *GhOPR11*, *GhAOS6* and *GhLOX3*, concurrent with root JA/JA‐Ile accumulation (Luo et al., 2019). Severe soil drying in rice (*Oryza sativa*) increased and decreased root expression of *OsAOC* and *OsOPR7* respectively, while root concentrations of OPDA, JA and JA‐Ile declined (Dhakarey et al., 2017). Although soil drying can stimulate expression of genes in the jasmonates biosynthesis pathway, gene expression analyses do not necessarily correlate with tissue jasmonate accumulation. Nevertheless, soil drying stimulated JA accumulation in multiple species including soybean (Castro‐Valdecantos et al., 2021) and tomato (Muñoz‐Espinoza et al., 2015). While ABA accumulation in drying soil parallels stress intensity, JA accumulation is characterised by early, transient increases (Wang et al., 2020). Soil drying increased endogenous concentrations of jasmonates (OPDA, JA and JA‐Ile) in roots, xylem sap and leaves of tomato (de Ollas et al., 2018) and root and leaf JA concentrations of soybean (Castro‐Valdecantos et al., 2021). Nevertheless, many studies do not find changes in jasmonates after drought stress exposure or even a decrease, with foliar JA and JA‐Ile concentrations decreasing with soil water content in hops, *Humulus lupulus* (Korovetska et al., 2016). Since accumulation of jasmonates may be transient, frequently measuring soil and plant water status and hormone quantification is necessary to determine soil moisture thresholds causing jasmonates to accumulate. If jasmonates loading into the xylem is maintained as the soil dries, decreased transpirational flow may cause jasmonates to accumulate in the xylem sap. Thus, it is necessary to evaluate the physiological significance of root‐supplied jasmonates.

### 2.6 Do root‐synthesised jasmonates affect shoot responses?

**FIGURE 2:**
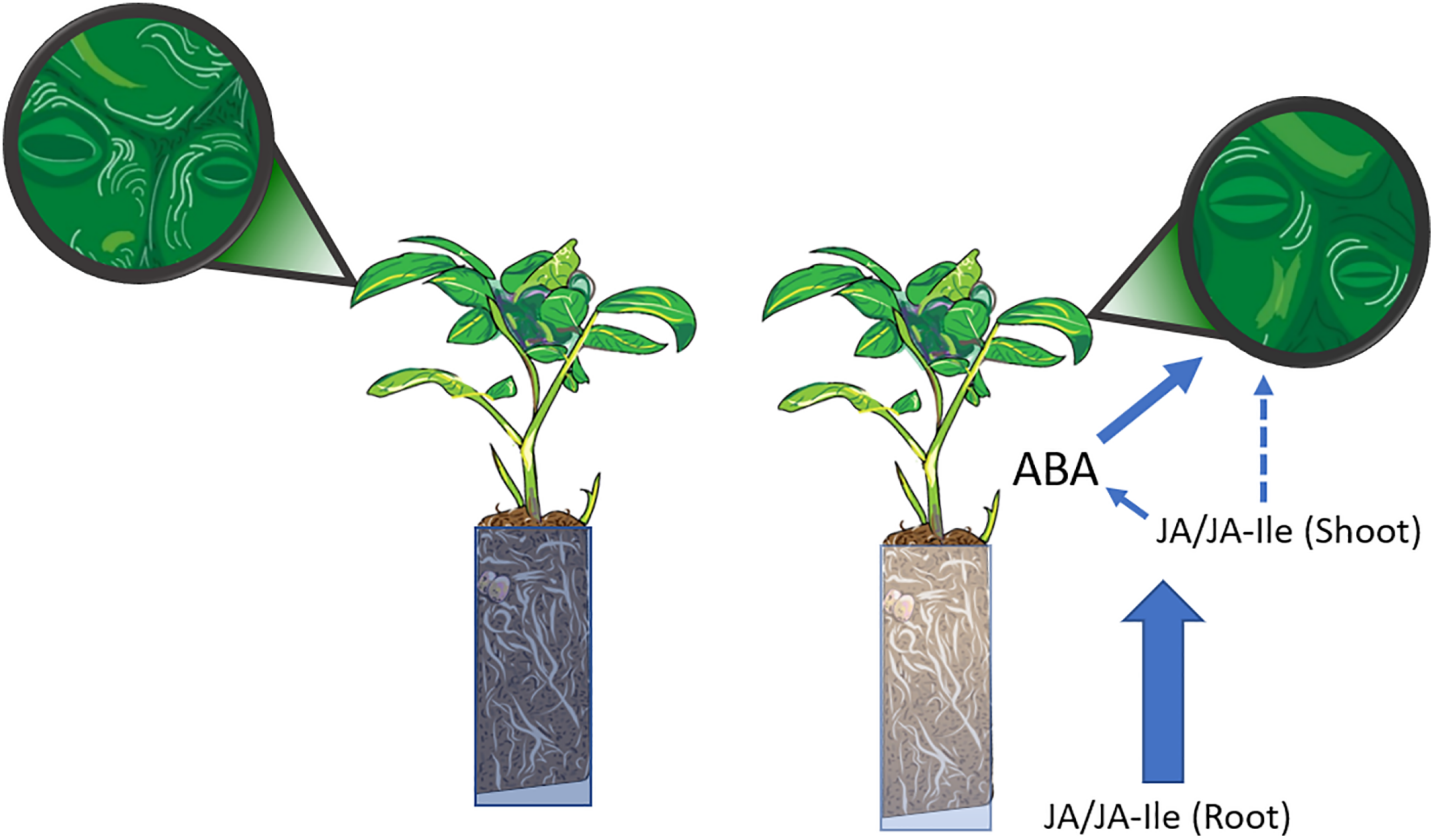
Increased jasmonates (JA/JA‐Ile) export from the root system as the soil dries stimulates shoot ABA accumulation, and has antitranspirant activity (dotted arrow), although much less than ABA. Artwork courtesy of Wallis Allen

Xylem‐supplied JA was sufficient to decrease transpiration of detached tomato leaves (de Ollas et al., 2018), even if its antitranspirant effects were less than those of ABA (Figure 2). Paradoxically, attenuating root export of jasmonates in the transpiration stream by grafting WT plants onto the *def‐1* rootstock (with compromised jasmonates synthesis but higher OPDA levels) decreased stomatal conductance (*g*_s_) of well‐watered plants, but did not alter stomatal sensitivity to drying soil (de Ollas et al., 2018), while grafting *def‐1* plants onto a WT rootstock promoted *g*_s_ of well‐watered plants. These unexpected rootstock‐mediated effects were attributed to differential export of OPDA from the rootstocks (low in WT, high in *def‐1*), a molecule with a greater antitranspirant activity than JA (Savchenko et al., 2014). Since the genetic nature of the *def‐1* tomato mutant has not been characterised, further grafting experiments are required with mutants further downstream of AOS and AOC to illustrate the importance of root‐supplied jasmonates in regulating plant water use.

### 2.7 Shoot‐to‐root jasmonates signalling

Impacts of soil water availability on root jasmonates accumulation may be partially attributed to their basipetal transport from the leaves, with foliar signals regulating root function. In cotton plants grown hydroponically with their roots split between unamended and PEG‐amended nutrient solution, girdling at the root‐shoot junction diminished JA/JA‐Ile concentrations and hydraulic conductance (*L*_p_) of the hydrated roots (Luo et al., 2019). Positive correlations between root JA status and *L*_p_ following exogenous MeJA treatment and in comparing the *def‐1* mutant with WT plants suggests that jasmonates promote root hydraulic conductance by upregulating aquaporins (de Ollas et al., 2018; Sanchez‐Romera, Calvo‐Polanco, et al., 2018). However, since jasmonates negatively regulate expression of some aquaporin genes and positively regulate others (Sanchez‐Romera, Ruiz‐Lozano, et al., 2018), conflicting observations of the impact of *def‐1* on *L*_p_ suggest that further measurements are necessary.

Interestingly, silencing foliar expression of the cotton JA biosynthesis genes *GhOPR11*, *GhAOS6* and *GhLOX3* decreased root jasmonates concentrations and Lp (Luo et al., 2019), again indicating substantial basipetal phloem transport of jasmonates. Likewise, girdling soybean plants at the root–shoot junction attenuated root JA accumulation in response to soil drying, even though girdling slightly increased root JA concentration of well‐watered plants (Castro‐Valdecantos et al., 2021). Similar to ABA, even if roots have the capacity to synthesise jasmonates, root JA accumulation partially depends on the shoots.

### 2.8 ABA–JA interactions

ABA and JA signalling pathways extensively interact in response to stress to co‐ordinate physiological responses (de Ollas & Dodd, 2016). Limited soil drying‐induced root JA accumulation in the ABA‐deficient tomato mutant *flacca* (compared with WT plants) was explained by decreased *AOC* and *OPR3* gene expression, while greater shoot JA concentration in *flacca* was explained by enhanced expression of these genes (Muñoz‐Espinoza et al., 2015). Jasmonate export from the root system enhanced soil‐drying induced shoot ABA accumulation (de Ollas et al., 2018; Figure 2). Downregulating the microRNA miR396 in tomato plants decreased transpiration by enhancing ABA accumulation (despite limited changes in NCED gene expression relative to non‐transformed plants) and enhancing JA‐Ile accumulation coincident with upregulated LOX gene expression (Fracasso et al., 2021). Thus, these two hormones seem to affect each other's synthesis, and further attention should be placed on understanding their metabolism.

In addition to influencing jasmonates biosynthesis, crosstalk between ABA and JA can affect sensitivity to fixed hormone concentrations hence modifying overall physiological response. Thus, JA can also modify sensitivity to ABA thereby enhancing its effects on stomatal closure or hydraulic conductivity. MeJA‐mediated regulation of stomatal closure is postulated to interact with ABA‐mediated regulation of Ca^2+^ signal transduction pathways (Förster et al., 2019), and both ABA and MeJA induce the formation of ROS and nitric oxide (NO) in guard cells (Hossain et al., 2011), which are involved in downstream signalling. Moreover, differential sensitivity to JA between strawberry cultivars explained different stomatal responses to drought despite a similar ABA response (Merlaen et al., 2020). This example illustrates the difficulty of assessing hormonal interactions, as cultivars and species can significantly differ in their hormone accumulation and sensitivity.

### 2.9 Strigolactones

While foliar sprays of grapevine with the synthetic SL GR24 indicate its antitranspirant action within hours of application in dicots, maintenance of leaf water status in such plants as the soil dries can result in greater stomatal conductance (Min et al., 2019), related to an attenuation of foliar ABA concentration. Nevertheless, both roots and shoots produce SL, and their biosynthesis has been reviewed in depth (Ruyter‐Spira et al., 2013). Interestingly, ABA and SL biosynthesis share a precursor (all‐trans‐ß‐carotene). In SL biosynthesis, this precursor is transformed into carlactone and 5‐deoxystrigol by the enzymes D27, CCD7 (MAX3 in *Arabidopsis*, RMS5 in pea), CCD8 (MAX4 in *Arabidopsis*, RMS1 in pea, D10 in rice), cytochrome P450 and possibly other enzymes (Ruyter‐Spira et al., 2013). Unlike the ABA biosynthesis genes, expression of *Sl*CCD7 and *Sl*CCD8 in tomato is considerably higher in roots and stolons than in leaves and flowers, suggesting below‐ground organs are the main site of SL biosynthesis (Visentin et al., 2016).

### 2.10 Long‐distance SL signalling

**FIGURE 3:**
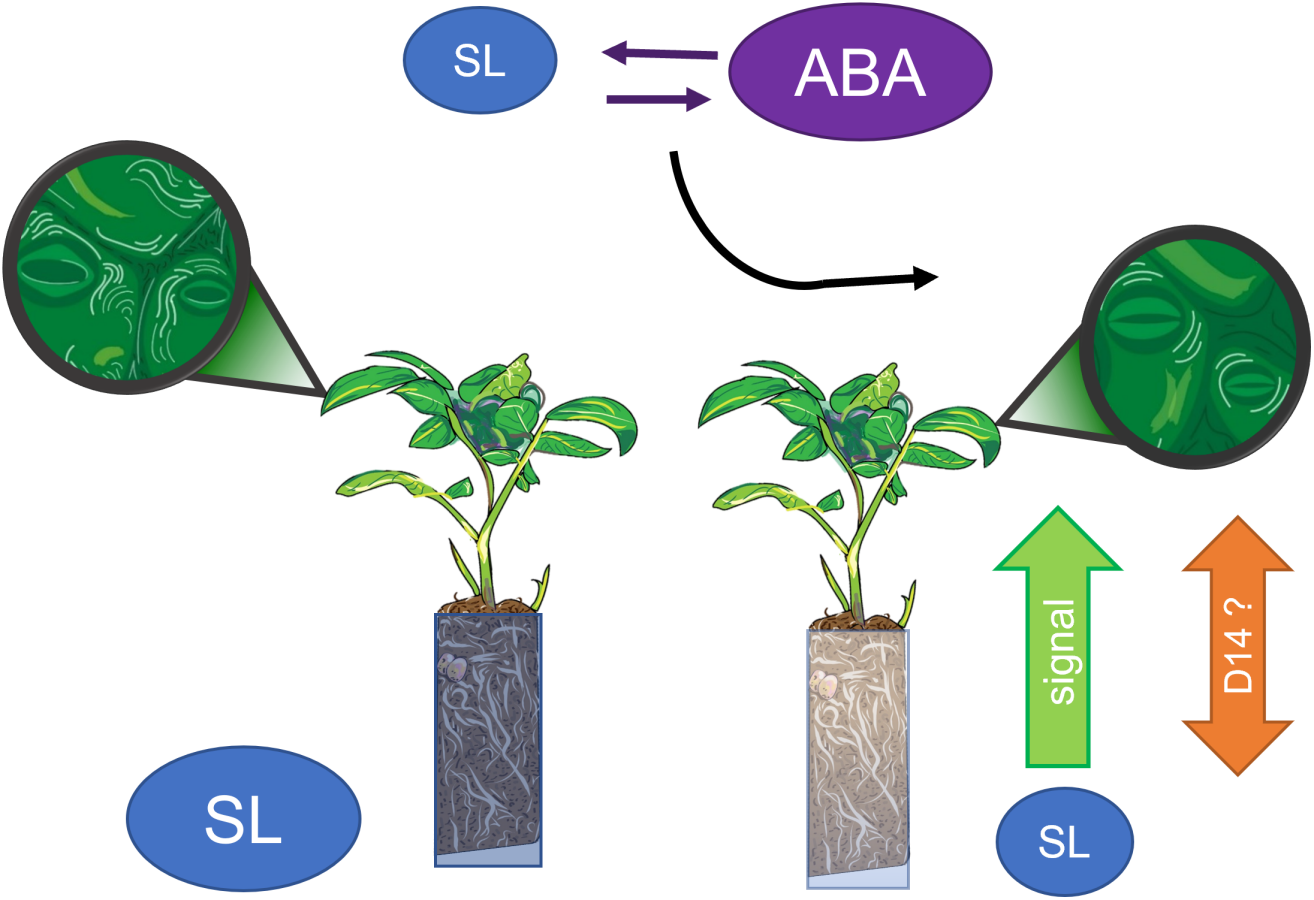
Decreased strigolactones (SL) biosynthesis in the root system as the soil dries triggers signals that interact with shoot SL and ABA accumulation to regulate stomatal closure, with the phloem‐mobile SL receptor D14 moving bidirectionally in some species to mediate SL effects. Artwork courtesy of Wallis Allen.

Grafting experiments in pea (Beveridge et al., 2000) and tomato (Hasegawa et al., 2018) showed that root‐synthesised SLs could phenotypically revert the branching phenotype of SL‐deficient shoots, indicating acropetal SL transport from the root. Unlike ABA, SL transport is unidirectional with no evidence of basipetal transport of SL from the shoot to the root (Kameoka & Kyozuka, 2018). PDR1, an ABC transporter identified in petunia, facilitates SL cell‐to‐cell transport, but is not expressed in the vasculature (Borghi et al., 2016). This indicates that other compounds in the SL biosynthesis and signalling pathway undergo long distance signalling, with methyl carlactone (reviewed in Borghi et al., 2016) and micro RNA (miR156, reviewed in Brun, 2020) suggested to be mobile. The D14 protein is a SL receptor that is phloem mobile in pea and can be transported bidirectionally across grafting junctions from root to shoot and from shoot to root, independently of SL transport (Kameoka & Kyozuka, 2018). A mobile receptor can enhance the efficacy of SL in the shoot and potentially function as a feedback mechanism to the root (Figure 3), indicating shoot SL status. However, in *Arabidopsis* D14 was not transported acropetally across a grafting junction (Chevalier et al., 2014) and D14 and KAI2 of the Karrikin signalling pathway both interact with the MAX2 receptor, which makes it difficult to differentiate effects of these two proteins (Borghi et al., 2016). Thus, SLs and their precursor carlactone are only transported acropetally, while the SL receptor D14 can be bidirectionally mobile, but interactions with ABA are not yet sufficiently understood, because they depend on many factors (Brun, 2020; Figure 3).

While SLs inhibit shoot branching in various species (Ruyter‐Spira et al., 2013), SL‐deficient mutants had increased stomatal conductance (Liu et al., 2015; Visentin et al., 2016). Stomatal density and aperture were increased in unstressed SL‐deficient and SL‐insensitive *Arabidopsis* (Lv et al., 2018; van Ha et al., 2014), but the SL insensitive *Atmax2* mutant only showed increased stomatal aperture compared with the WT as the soil dried out (Bu et al., 2014). Nevertheless, these phenotypes could explain lower stomatal conductance (*g*_s_) of WT *Arabidopsis* than SL‐deficient mutants (Kalliola et al., 2020). In drying soil, *g*_s_ of SL‐deficient tomato and *Lotus* is higher than in WT plants, until severe drought stress caused almost complete stomatal closure (Liu et al., 2015; Visentin et al., 2016). However, tomato WT shoots grafted onto SL deficient rootstocks had a lower *g*_s_ than the WT self‐graft under well‐watered conditions and were hypersensitive to ABA, with the authors postulating that decreased SL transport from the roots under drought stress leads to increased SL production in the shoot and hence higher sensitivity to ABA (Visentin et al., 2016). Interestingly, SL content in roots of lettuce and tomato decreases with decreasing soil water availability (Ruiz‐Lozano et al., 2016; Visentin et al., 2016). However, studies with reciprocal grafts of species other than tomato are required to support this hypothesis. Taken together, SLs are root‐derived signals that decrease stomatal conductance, suggesting they may act as root‐to‐shoot signals of drying soil and interact with ABA.

### 2.11 ABA–SL interactions

SL deficient tomato shoots had 33% lower ABA levels than the WT under severe drought stress (Visentin et al., 2016), ABA levels of SL‐deficient *Lotus* plants were similar to the WT under control conditions and osmotic stress (Liu et al., 2015), while shoots of SL‐deficient rice plants had higher or lower ABA levels (by no more than 10%) than the WT depending on the lesion in the SL biosynthesis pathway (Haider et al., 2018). Further studies quantifying variation in ABA levels as a function of soil and plant water status in diverse SL‐deficient mutants from different species seem necessary to resolve these contradictions. The ABA‐deficient tomato mutants *notabilis*, *flacca* and *sitiens* had lower SL levels in root exudates than WT plants (López‐Ráez et al., 2008), indicating a positive interaction between ABA and SL in tomato, but this interaction may depend on the species. To our knowledge, no experiments have directly compared stomatal closure of SL‐deficient and ABA‐deficient mutants in response to soil drying. Furthermore, reciprocally grafting ABA‐ and SL‐deficient mutants could determine the relative importance of acropetal and basipetal transport of SL and ABA in root and shoot function.

Since SL and ABA both target the anion channel SLAC1 to induce stomatal closure (Lv et al., 2018), they likely interact in drought stress signalling. Guard cells of SL‐deficient and SL‐insensitive *Arabidopsis* are less sensitive to exogenous ABA and a number of ABA biosynthesis and signalling genes were downregulated in the SL‐insensitive mutant compared with the WT (Bu et al., 2014; van Ha et al., 2014). Excised leaves of SL deficient mutants in *Arabidopsis* and tomato were reportedly less sensitive to exogenous ABA (Bu et al., 2014; van Ha et al., 2014), but it is unclear how this interaction is mediated. Further research is needed to understand whether SL impacts on stomatal conductance vary between species and its possible interactions with other signals. While SLs fulfil the criteria of a genuine root‐to‐shoot signal, their relative importance in regulating stomatal responses to drying soil would perhaps be best addressed by studying the water relations of SL‐ and ABA‐ deficient or ‐insensitive double mutants.

## 3 CONCLUSIONS

Since ABA is a potent antitranspirant, able to elicit stomatal closure in isolated epidermis at ABA concentrations lower than those detected in the xylem sap of well‐watered plants (Trejo et al., 1995), many studies have focused on quantifying its levels throughout the plant (Castro et al., 2019). Increasing recognition of the importance of other signalling molecules (de Ollas et al., 2018; Malcheska et al., 2017; Visentin et al., 2016), especially following a paradigmatic shift that ABA acts as a shoot‐to‐root rather than root‐to‐shoot signal, has prompted some to question the basis of root‐to‐shoot chemical signalling altogether (Tardieu, 2016). Prompt stomatal responses to fluctuations in leaf water status (e.g. caused by changes in evaporative demand) which may involve guard‐cell autonomous ABA biosynthesis, would seem to obviate the need for root‐to‐shoot signalling altogether. Nevertheless, opportunities to use superior rootstocks in horticultural crops, even if rootstock hydraulic conductance mediates hydraulic signalling mechanisms, should stimulate a better fundamental understanding of the signalling systems reviewed herein. Perturbing a single hormone biosynthesis gene in the rootstock (overexpressing NCED) caused multiple changes in root gene expression (e.g. downregulation of JA biosynthesis genes) that partially explained variation in shoot hormone status (e.g. Martínez‐Andújar et al., 2021). Understanding how these hormones interact in specific tissues (rootstock or scion), and integrate various environmental cues that regulate shoot growth, leaf gas exchange, root growth and root hydraulic conductance, remains a challenging objective. Nevertheless, the prospects of enhancing commercial production by judicious rootstock selection (e.g. 50% yield increment by grafting onto NCED overexpressing rootstocks—Martínez‐Andújar et al., 2021) should stimulate biotechnological exploitation of the signalling systems discussed herein.

## Data Availability

Data sharing is not applicable to this article as no new data were created or analyzed in this study.
